# Phosphodiesterase 4D, miR-203 and selected cytokines in the peripheral blood are associated with canine atopic dermatitis

**DOI:** 10.1371/journal.pone.0218670

**Published:** 2019-06-21

**Authors:** Jeffrey Koury, Ana Ramirez, Chen Xie, Jerry Harb, Charli Dong, Chad Maki, Tom Ramos, Fari Izadyar, David Clark, Yvonne Drechsler, Gagandeep Kaur, Jijun Hao

**Affiliations:** 1 Graduate College of Biomedical Sciences, Western University of Health Sciences, Pomona, California, United States of America; 2 College of Science, California State Polytechnic University, Pomona, California, United States of America; 3 College of Veterinary Medicine, Western University of Health Sciences, Pomona, California, United States of America; 4 Animal Dermatology Clinic, Pasadena, California, United States of America; 5 VetCell Therapeutics, Santa Ana, California, United States of America; INSERM, FRANCE

## Abstract

Canine Atopic Dermatitis (AD) is a common complex and multifactorial disease involving immune dysregulation, genetic predisposition, skin barrier defects, environmental factors and allergic sensitization. To date, diagnosis of canine AD relies on a combination of patient history, clinical examination, allergy testing and response to diet trials/therapies with no reliable biomarkers available to distinguish AD from other diseases with similar clinical presentations. A handful of studies to identify potential biomarkers in the peripheral blood of AD dogs and healthy controls have been performed with some showing inconsistent and contradictory results. In this study, we, for the first time, report statistically significant increases in expression of phosphodiesterase 4D (PDE4D) gene in peripheral blood mononuclear cells (PBMCs) and miR-203 in plasma from AD dogs compared to healthy controls. In addition, we report a statistically non-significant change of the CD4^+^/CD8^+^ ratio, a dramatic decrease of three gene markers (PIAS1, RORA and SH2B1) as well as a panel of differential expression of cytokines in AD dogs in comparison to the healthy controls. Our study provides important insight into the complexities of canine AD, and further studies to verify the specificity of these findings for canine AD at a larger-scale are warranted.

## Introduction

Canine atopic dermatitis (AD) is a common genetically predisposed inflammatory and pruritic allergic skin disorder in dogs worldwide [1]. Although the pathogenesis remains elusive, epidermal barrier dysfunction and immune dysregulation following allergen exposure are believed to be implicated in the development of canine AD [2–4]. In canine AD, allergic skin inflammation is in part attributed to the diminished skin barrier function and the increased Type 2 Helper T (Th2) cell response. In the early acute phase, Th2 responses play predominant roles and induce production of IgE antibodies and various pro-inflammatory cytokines including IL-4, IL-5, IL-13 and IL-31 to promote the development of humoral immunity and hypersensitivity response during sensitization [5–7]. In contrast, in the chronic phase of canine AD, Type 1 Helper (Th1)-dominated inflammatory reactions promote secretion of cytokines such as interferon-γ (IFN-γ) [5, 8, 9].

To date, diagnosis of canine AD relies on a combination of patient history, clinical examination, allergy testing and response to diet trials/therapies, and no reliable biomarkers are available to distinguish canine AD from other similarly presenting diseases such as food allergies, pyoderma, flea allergy dermatitis and malassezia dermatitis [10]. To address this issue, efforts have been made by examining specific immune cells, cytokines and genes from the lesion and non-lesion skin biopsies or peripheral blood of both AD dogs and healthy controls [11–14]. In contrast to obtaining dog skin biopsies for diagnostic studies, collection of blood samples is more convenient and less invasive for dogs, and results from blood studies reflect the overall immune responses and are more reliable as variability among different lesions is minimized. Nevertheless, to date, only limited studies with some contradictory results have been reported in the blood-based biomarker profiling in canine AD. For instance, Tarpataki et al. reported an increase of CD4^+^/CD8^+^ ratio of lymphocytes while two other groups showed opposite results in serums of AD dogs, and some reported no differences in the CD4+/CD8+ ratio [11–13, 15].

Phosphodiesterase 4 (PDE4) is a predominant enzyme degrading cyclic adenosine monophosphate (cAMP), an intracellular second messenger known to regulate pro- and anti-inflammatory activities, in most immune cells, and PDE4 is also involved in a variety of epithelial functions including skin barrier protection [16–18]. Human clinical studies have shown that inhibition of PDE4 is beneficial to children and adults with AD [19–25]. In addition, PDE4 inhibitor arofylline also improves pruritus in 70% of dogs with AD after 4 weeks of treatment, but unfortunately, this arofylline benefit to AD dogs is compromised by the prominent adverse event—vomiting [26]. To date, gene expression of PDE4s in peripheral blood mononuclear cells (PBMCs) of both humans and dogs with AD has not been reported. In addition, microRNAs (miRNAs), which interfere with mRNA translation, are becoming increasingly recognized as powerful biomarkers for various diseases [27]. Recently, two miRNAs, miR-203 and miR-483, have been shown to be upregulated in serum of children with AD, but no miRNAs have been studied in canine AD which share many characteristics similar to their human counterparts [4, 28, 29]. Here, we first examine expression levels of all four PDE4 gene isoforms, miR-203 and miR-483 in AD dogs compared to healthy controls. In addition, we also verify the CD4^+^/CD8^+^ cell ratio, total circulating IgE antibodies, expression levels of the previously reported-AD associated genes (PIAS1, RORA, SH2B1) and a panel of cytokines (IL-4, IL-10, IL-13, IL-31, IFN- γ, TGF- β1, TNF-α) in AD dogs compared to healthy controls.

## Materials and methods

### Animals

This study complies with institutional guidelines on the use of animals in clinical research and was approved by IACUC committee of Western University of Health Sciences (Pomona, CA, USA). Written consent forms were obtained from owners for this study. A total of nine client-owned AD dogs (six neutered males and three spayed females) with naturally occurring AD were enrolled in this study from August 2017 to March 2018 (**S1 Table and S2 Table**). The AD dog breeds as reported by owners include Miniature Pinscher mix, Golden Retriever, German Shepard, Shih Tzu, Great Dane, Cocker Spaniel, Boxer, Poodle and Terrier Mix. In addition, another eight client-owned healthy dogs without AD (4 neutered males, 3 spayed females, and 1 intact female) were enrolled in this study as controls. The healthy dog breeds reported by owners include Rat Terrier, Chihuahua, Mixed, Pitbull mix, Plott Hound, and Australian Cattle Dog cross.

### Inclusion criteria for AD dogs

Clinical diagnosis of AD was based on detailed interpretation of patient history, clinical signs, and exclusion of other possible skin dermatosis that can present as AD. History included the age of onset, alesional prior to clinical signs, primary lesions such as erythema or papules, distribution of lesions, history of ear infections, history of persistent pruritus that worsens with secondary infections, and is aggravated by allergies-seasonal or food related. Clinical signs were as outlined in Hensel et al [30]. These included: pruritus, erythema, papules, or self-trauma to the face, concave aspect of pinnae, ventrum, axillae, inguinal region, perineal region, and distal extremities. Flea combing, skin scrapings, skin cytologies and elimination diet trials were performed. These are in accordance with the guidelines developed by the International Committee for Allergic Diseases in Animals (ICADA) diagnosis of canine AD [31]. Clinical presentation and skin scrapings were used to rule out skin parasites such as scabies and demodicosis. Initial negative scrapings were followed by maintenance on oral afoxolaner, which has been shown to be curative for demodex and sarcoptes mites [31, 32]. Strict elimination diet trial with hydrolyzed protein diet were undertaken for at least 8 weeks duration, re-examined, then followed by challenge with original diet at the end of the trial. Patients in the AD group were over one year of age, with a body condition score of at least 4 on a 9-point scale. Underlying systemic diseases were ruled out through thorough physical examinations and serum chemistry and hematology analyses. Participants were current on ectoparasite control and prevention during the study.

### Exclusion criteria for AD dogs

Clinical evidence of ectoparasite infestations (flea allergy dermatitis, scabies etc.), bacterial or fungal cutaneous infections, food allergies, and seasonality of the cutaneous condition resulted in exclusion from the study. Ongoing treatment with anti-inflammatory or immunosuppressive medications also resulted in exclusion, unless appropriate weaning times were followed. Weaning or withdrawal times were observed for a period of 8 weeks for glucocorticoids (topical, injectable, oral), Cytopoint, Apoquel, 4 weeks for cyclosporine, and 2 weeks for oral antihistamines or non-steroidal anti-inflammatory drugs.

### Inclusion criteria for healthy dogs

Participants that were greater than 1 year of age, had a body condition score of at least 4 on a 9-point scale, with no history or clinical signs of pruritus or immune modulating disease conditions were enrolled in the study. These dogs were examined at their respective DVM’s and had normal physical exam findings, complete blood counts and blood chemistries within the last 12 months. Only dogs that were current on ectoparasite control, did not have history or clinical signs of pruritus, did not receive any medications for conditions related to pruritus, were not treated for any immune related conditions and did not receive any immune modulating drugs over the course of last 12 months were included in this group.

### Flow cytometry

PBMCs were isolated from whole blood collected in EDTA vacutainers. In a separate tube, 2mL of whole blood was diluted with 6 mL of Phosphate Buffered Saline. Diluted whole blood was layered on top of 2mL of Ficoll-Paque PLUS (GE Healthcare Catalog #17-1440-02) and centrifuged at 2500 rpm for 25 minutes (no brake). The PBMC interphase was collected. Red blood cells (RBCs) were lysed with 1X RBC Lysis Buffer (BioLegend Catalog #420301) followed by spinning and resuspension in the cell staining buffer (BioLegend Catalog #420201). Antibody staining was conducted using Anti-dog CD3 Clone CA17.2A12:FITC, CD4 Clone YKIX302.9:RPE, CD8 YCATE55.9:Alexa Fluor647 (Bio-Rad) and staining with 10uL of isotype control Bio-Rad MSE IgG1:FITC/RAT IgG2a:RPE/RAT IgG1:Alexa Fluor647 (Bio-Rad Catalog #TC023). Cells were resuspended in 400ul of cell staining buffer, stained with 5uL of 7-AAD Viability Dye (BioLegend Catalog #420404) and analyzed on the BD Accuri C6 Flow Cytometer.

### Serum ELISA analysis

Serum ELISA analysis was carried out according to the manufacturer’s protocols and the following ELISA kits were used for this study (Table 1).

**Table 1:** ELISA kits information used for measurement of canine cytokines.

Canine Cytokine	Company	ELISA Kit	Catalog Number
IL-4	NeoBioLab	Canine IL4 ELISA Kit	CI0014
IL-10	R&D Systems	Canine IL10 Quantikine ELISA Kit	CA1000
IL-13	NeoBioLab	Canine IL13 ELISA Kit	CI0043
IL-31	NeoBioLab	Canine IL31 ELISA Kit	CI0041
IFN- γ	R&D Systems	Canine IFN- γ Quantikine ELISA Kit	CAIF00
TGF- β1	R&D Systems	Mouse/Rat/Porcine/Canine TGF- β1 Quantikine ELISA Kit	MB100B
IgE	Abcam	Canine IgE ELISA Kit	Ab157700
TNF-α	R&D Systems	Canine TNF-α Quantikine ELISA Kit	CATA00

### RNA extraction and real-time PCR

RNA was extracted from PBMCs by using the RNeasy Mini Kit (Qiagen, Catalog #74104) according to manufacturer’s protocol with an additional DNAse I digestion step. cDNA was synthesized from the extracted RNA using the High Capacity cDNA Reverse Transcription Kit following the manufacturer’s instructions (Applied Biosystems, Catalog #4368814). The reactions were performed in triplicate on Bio-Rad CFX connected Real-Time PCR system. Canine glyceraldehyde-3-phosphate dehydrogenase (GAPDH) gene was used as an internal control. The following primer sets were designed and used for real-time PCR (Table 2):

**Table 2:** Primer sequences used for RT-PCR.

Gene	Forward	Reverse
Canine PIAS1	5’-TGGAGTTGATGGATGCTTGAG-3’	5’-GGACACTGGAGATGCTTGAT-3’
Canine RORA	5’-AAGGCTGCAAGGGCTTTTTC-3’	5’-CTGCGTACAAGCTGTCTCTT-3’
Canine SH2B1	5’-CGTCCTCACTTTCAACTTCCA-3’	5’-GACACGACATAGCTGACAAGA-3’
Canine PDE4A	5’-GGTGAAGACAGATCAAGAGGAG-3’	5’-GTGCAAGCTGTTGTGGTAAG-3’
Canine PDE4B	5’-GAGCTGGAAGACCTGAACAA-3’	5’-CAGCGTGTAGGCTGTTATGA-3’
Canine PDE4C	5’-GGTCTCCAACCAGTTTCTCATC-3’	5’-TCCAAGGCTAGTCACCTTCT-3’
Canine PDE4D	5’-AATCACAGGTGGGCTTCATAG-3’	5’-CACTGCAGCTAGTGTCTTCTT-3’
Canine GAPDH	5`-GGAGAAAGCTGCCAAATATG-3’	5’-ACCAGGAAATGAGCTTGACA-3’

### MicroRNA expression by real-time PCR

Whole blood collected with EDTA coated tubes was spun down and the plasma supernatant containing miRNA was collected. Extraction of miRNA was performed by following the protocol outlined in miRNeasy Serum/Plasma Kit (Qiagen, Catalog #217184) and the miRNeasy Serum/Plasma Spike-In Control was used (Qiagen, Catalog #219610). The reverse transcription was conducted by following the protocol of “Taqman Small RNA Assays” (Applied Biosystems, Catalog # 4366596). TaqMan real-time PCR assays were performed in triplicate with Bio-Rad CFX connected Real-Time PCR system according to the manufacturer's instructions. Data were normalized to the internal control miR-39. The following TaqMan probe and primer sets (ThermoFisher) were used: miR-39 (RT 000200), miR-203 (RT 000507) and miR-483 (RT 002560).

## Results

### PDE4D gene expression is significantly upregulated in AD dog PBMCs

**Fig 1:**
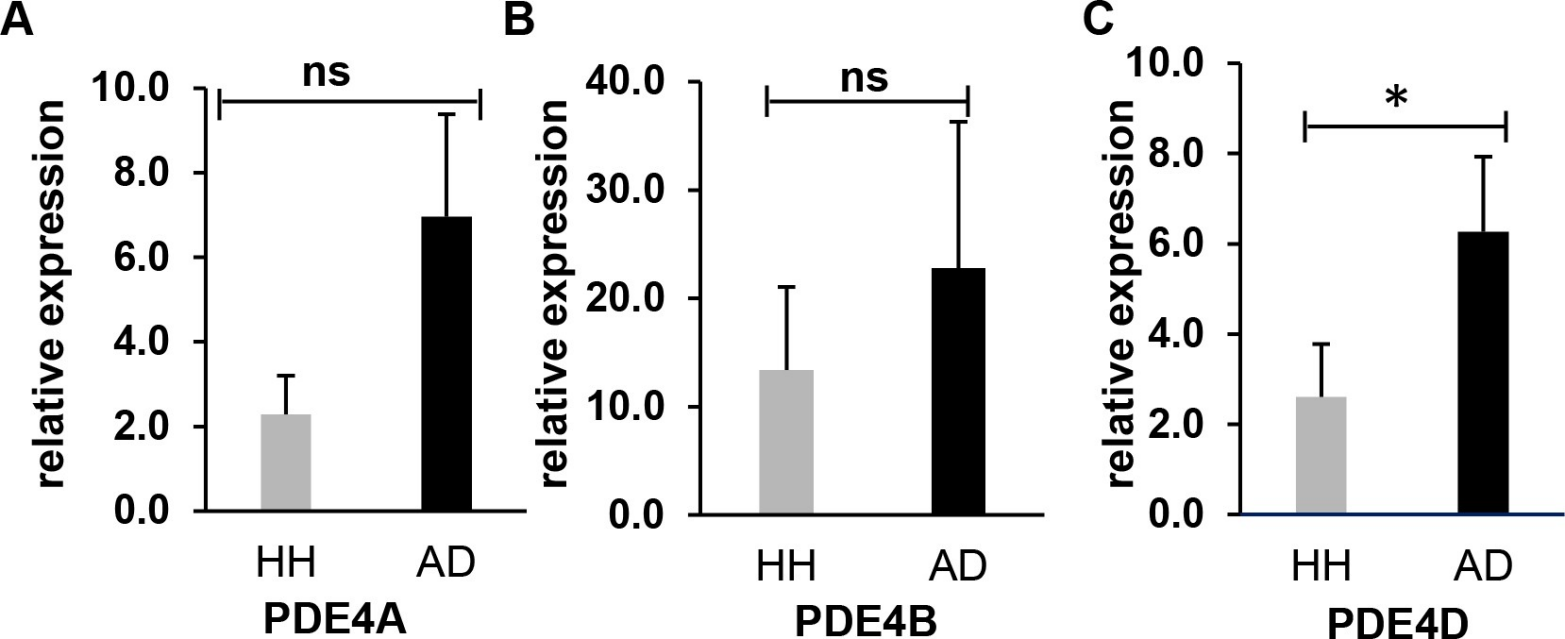
RT-PCR results show gene expression of PDE4 in AD dogs compared to the healthy controls. Expression levels of PDE4A **(A)**, PDE4B (**B**) and PDE4D (**C**) were elevated in the PBMCs of canine AD dogs in comparison with those of the healthy (HH) controls. Canine GAPDH gene was used as an internal control. Each bar is representative of a triplicate experiment (Healthy: n = 8, Atopic: n = 9). Non-parameter test; ns–Not Significant, *P < 0.05. RT-PCR results represent relative expression of AD dogs normalized to that of the health controls.

Multiple studies have shown that inhibition of PDE4 is beneficial to canine AD [19–22]. However, gene expression of PDE4s in PBMCs of canine AD has not been reported. We therefore examined all four PDE4 isoforms to verify whether any of these isoforms may be a potential marker(s) for canine AD. Blood samples were collected from eight healthy dogs and nine AD dogs, and RNA was then extracted from PBMCs of these blood samples. The RT-PCR results indicated that three out of four PDE4 isoforms (PDE4A, PDE4B and PDE4D) were upregulated in the AD samples whereas PDE4C gene was not detectable in both AD and heathy dog samples (**Fig 1**). Particularly, PDE4D gene expression in AD samples showed statistically significant upregulation by approximately 2.4-fold in comparison to that of the healthy control samples (p<0.05) (**Fig 1C**). Though the gene expression levels of PDE4A and PDE4B were also elevated in AD samples compared to the healthy control samples, both of the increases were not statistically significant. In summary, PDE4D may be a potential marker for AD dogs.

### MiR-203 and miR-483 are upregulated in AD dog plasma

**Fig 2:**
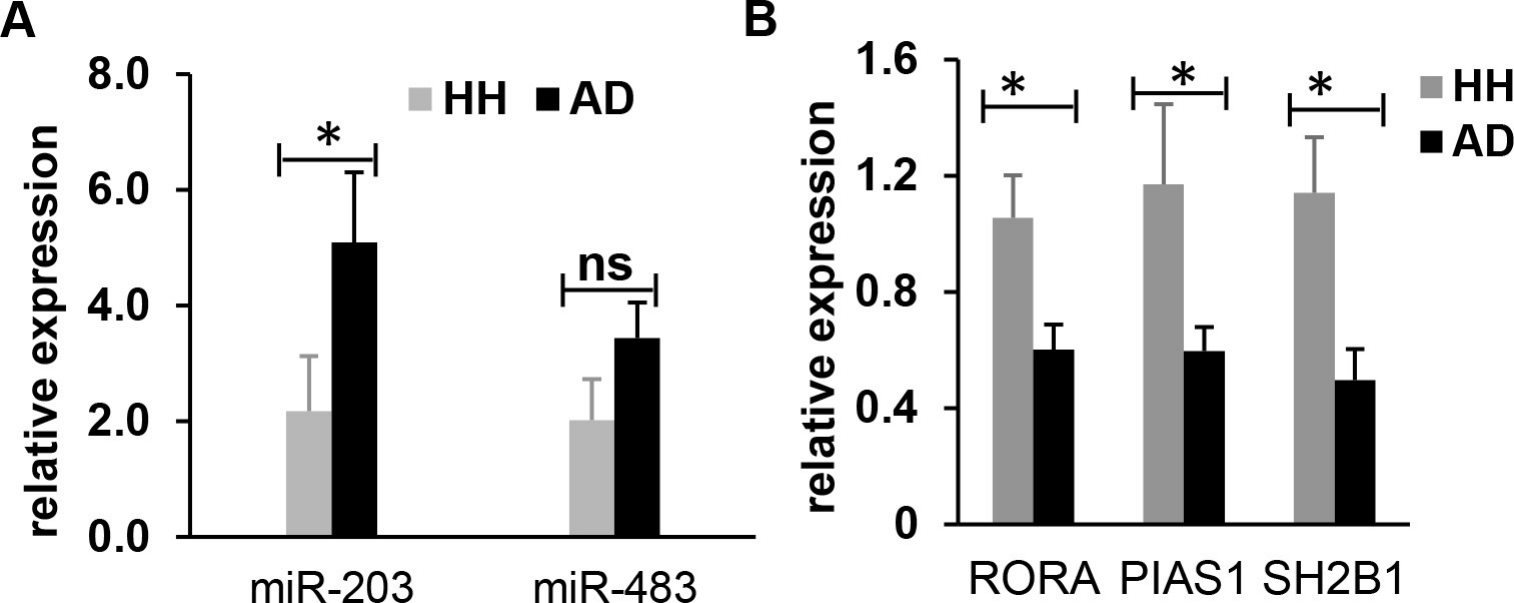
RT-PCR results show elevated expression of miR-203 and miR-483 and decreased expression of the specific genes (PIAS1, RORA and SH2B1) in canine AD dogs compared to the healthy controls. (**A**) Expression levels of miR-203 and miR-483 were elevated in the plasma of canine AD dogs by approximately 2.5-fold and 1.6-fold respectively in comparison with those of the healthy controls. The canine miR-39 was used as the internal control. (**B**) Expression levels of the three specific genes (PIAS1, RORA and SH2B1) were significantly downregulated in PBMCs of the canine AD dogs compared to the healthy dogs. Canine GAPDH gene was used as an internal control. Each bar is representative of a triplicate experiment for each patient (Healthy: n = 8, Atopic: n = 9). Non-parameter test; ns–Not Significant, *P < 0.05. RT-PCR results represent relative expression of AD dogs normalized to that of the health controls.

The circulating miR-203 and miR-483 were previously shown to be upregulated in human children AD sera [28]. However, to date, no study of miRNAs in AD dogs has been reported. As dog AD has many similar characteristics with its human counterpart, we examined miR-203 and miR-483 expression changes in the plasma of both AD dogs and healthy controls [4, 29]. Blood samples were collected from eight healthy dogs and nine AD dogs, and their plasma was prepared, followed by miRNA extraction. RT-PCR reactions were conducted to quantify expression of miR-203 and miR-483, and miR-39 was used as the internal control. In agreement with the previous report in human AD [28], our results showed elevated expression levels of miR-203 and miR-483 by approximately 2.5-fold (P = 0.036) and 1.6-fold (statistically not significant P = 0.093) respectively in the plasma of AD dogs in comparison with those of the healthy controls (**Fig 2A**). This result suggests that miR-203 may be a possible biomarker for AD of both humans and dogs.

### Verification of PIAS1, RORA and SH2B1 gene downregulation in the PBMCs of AD dogs

A single study has previously shown that expression levels of three genes (PIAS1, RORA and SH2B1) in PBMCs are down-regulated in the PBMCs of AD dogs [11]. To validate this result, we performed RT-PCR reactions to examine these gene expressions in PBMCs of both the AD dogs and healthy controls. In agreement with the previous report [11], our results confirmed that expression levels of all three gene (PIAS1, RORA and SH2B1) were significantly downregulated in the PBMCs of the AD dogs by approximately 1.5-fold for RORA gene (P = 0.015) and 2-fold for both genes of PIAS1 (P = 0.036) and SH2B1 (P = 0.011) in comparison with the healthy dogs (**Fig 2B**).

### The CD4^+^/CD8^+^ ratio of T lymphocytes, cytokines and total IgE in AD dogs

**Fig 3:**
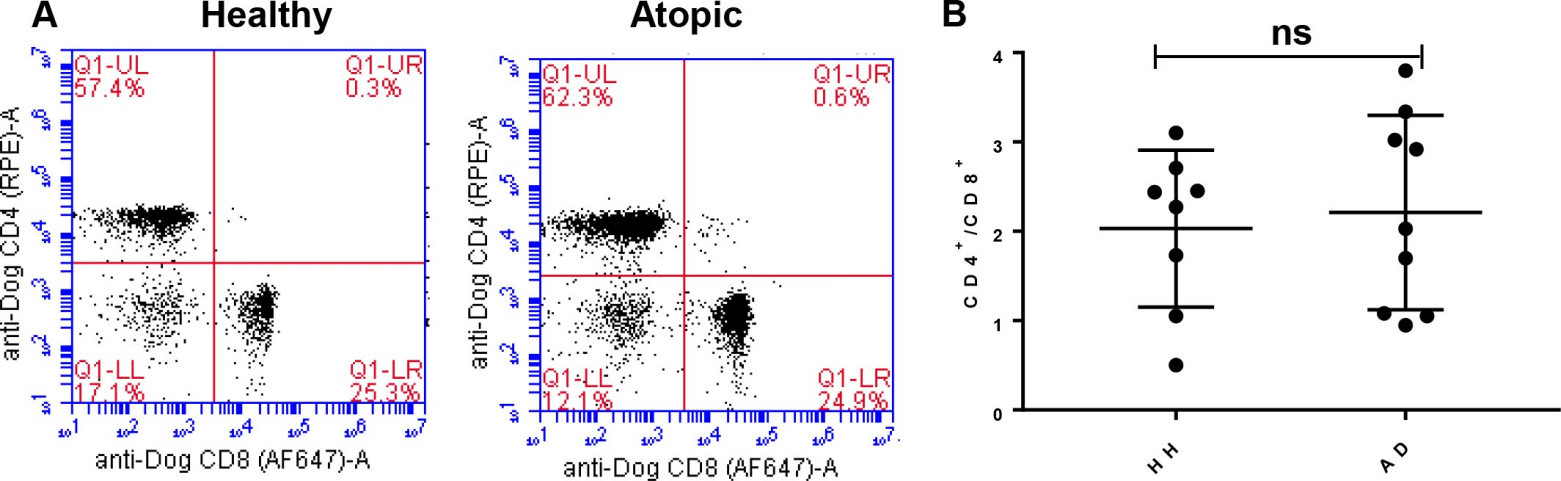
Analysis of CD4^+^ T cell compared to CD8^+^ T cells in healthy vs atopic canines. **(A)** CD4 vs CD8 flow plot from PBMCs of 1 healthy canine and 1 atopic canine. All plots were gated on lymphocytes and CD3^+^ Cells. Dead cells were excluded by 7-AAD. **(B)** Comparison of CD4^+^/CD8^+^ T Cell ratios between healthy canines (n = 8) and atopic canines (n = 9). The mean CD4^+^/CD8^+^ ratio of healthy canines was 2.031 ± 0.3105 compared to 2.21 ± 0.3626 for atopic canines (results expressed as mean ratio ±SEM). Non-parameter test; P = 0.7631 and not significant.

T lymphocytes are critical for the development and regulation of cell-mediated immune responses. An elevation of CD4^+^/CD8^+^ ratio of T lymphocytes has been shown to be associated with AD dogs whereas others argued against this with contradicting results [11–13]. In addition, one study stated no differences of the CD4+/CD8+ ratio between AD and healthy dog samples [11–13, 15]. To clarify, we compared the CD4^+^/CD8^+^ ratio of T lymphocytes in the PBMCs of AD dogs and healthy controls by flow cytometry analysis. Our data indicates a modest increase in CD4^+^ T Cells in the AD dogs’ samples in comparison with the healthy controls **(Fig 3A)**, resulting in a slight elevation of the CD4^+^/CD8^+^ ratio in AD dogs (2.388 ± 0.3747) vs. healthy controls (2.101 ± 0.2826) **(Fig 3B)**. Nevertheless, this increase of CD4^+^/CD8^+^ ratio of T lymphocytes associated with canine AD was not statistically significant (P = 0.7631).

**Fig 4:**
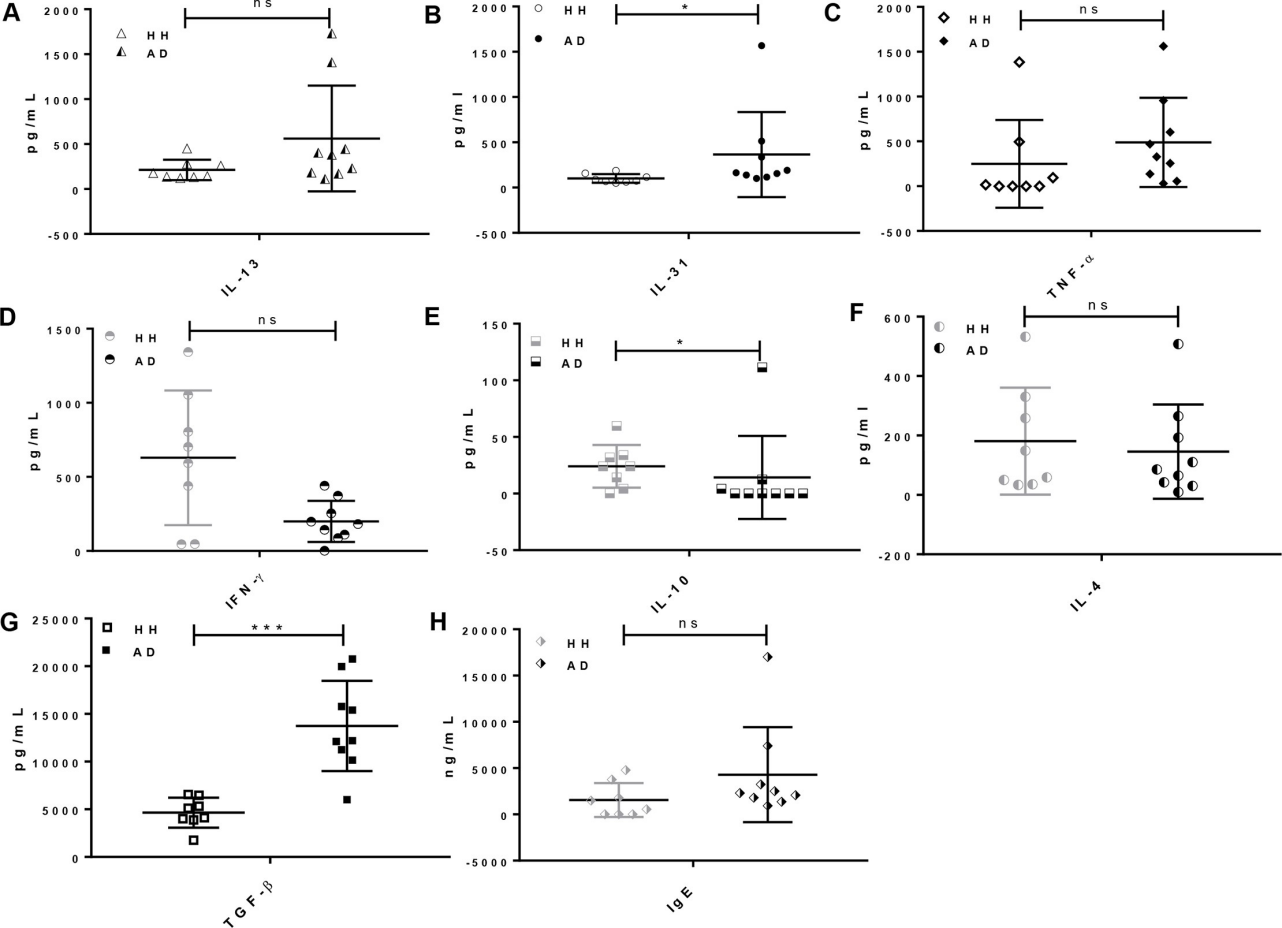
Cytokine profiles of healthy vs atopic canines. Serum was isolated from whole blood extracted from either healthy or AD dogs. Expression of a multitude of cytokines including **A)** IL-13, **B)** IL-31, **C)** TNF-α, **D)** IFN-γ, **E)** IL-10, **F)** IL-4, **G)** TGF- β1 and H) IgE were analyzed via ELISA. Each bar is representative of a duplicate experiment for each patient (Healthy: n = 8, Atopic: n = 9). Non-parameter test; NS–Not Significant, *P < 0.05, **P < 0.01, ***P < 0.001.

As canine AD is an inflammatory related-skin disease, we next examined a panel of cytokines of including TH2 cytokines (IL-4, IL-13 and IL-31), TH1 cytokine IFN-γ, anti-inflammatory cytokines (IL-10 and TGF- β1), and pro-inflammatory cytokine TNF-α by ELISA. Consistent with the previous reports [11, 32], inflammatory cytokines IL-13, IL-31 and TNF-α were significantly elevated (**Fig 4A, 4B and 4C** statistically non-significant for IL-13 and TNF-α) whereas the pro-inflammatory cytokine IFN-γ and anti-inflammatory cytokine IL-10 were dramatically decreased in AD patient sera **(Fig 4D and 4E)**. In addition, expression of IL-4, which induces Th2 cell differentiation and B-cell class switching to IgE, was barely changed (statistically non-significant) in AD patient sera as compared to the healthy controls (**Fig 4F**). This result is similar with the former report of IL-4 expression in canine AD patient plasma [11]. In addition, previous reports about TGF-β1 expression in canine AD are controversial. For instance, Fedenko et al. showed a significant elevation of TGF-β1 in AD patient blood samples compared to their healthy controls, whereas other studies reported opposite results [5, 11, 33]. Our study indicates that TGF-β1 expression in the canine AD sera is elevated by approximately 2.8-fold in comparison with the healthy controls (**Fig 4G**). Finally, the total serum IgE level displays no significant difference in the sera of AD dogs and the healthy controls which is in agreement with previous reports [34, 35] (**Fig 4H**).

## Discussion and conclusions

AD is a common disease in dogs, and the pathogenesis of canine AD has not been fully understood [1]. To date, diagnosis of canine AD relies on a combination of patient history, clinical examination, allergy testing and response to diet trials/therapies, and reliable AD specific biomarkers are lacking. Here, we assessed all four PDE4 isoforms, specific miRNA expression, expression levels of genes associated with canine AD, the CD4+/CD8+ ratio of T lymphocytes, and a panel of cytokines in peripheral blood of both canine AD dogs and healthy controls. We, for the first time, report statistically significant gene expression increase of PDE4D in PBMCs and miR-203 in sera from AD dogs. PDE4D catalyzes hydrolysis of cAMP, an intracellular signal that plays an important role in regulation of secretion of inflammatory mediators through activation of protein kinase A [16, 36, 37]. Recent studies indicated that cAMP is involved in negative regulation of Th1 cells and M2 polarization of macrophages [38, 39]. As a shift to a Th2 response is observed in the development canine AD, PDE4 expression might be involved in this process. Our result of the PDE4D gene expression increase is well aligned with previous studies that PDE4 inhibitors are beneficial to both humans and dogs with AD [19–26]. Nevertheless, all current available PDE4 inhibitor drugs competitively target the PDE4 catalytic domain, and are associated with severe emesis. Allosteric PDE4 inhibitors that target non-catalytic domain are believed to overcome the side effect of the competitive PDE4 inhibitors [40–42]. We have previously identified a novel allosteric PDE4 inhibitor, Eggmanone, which specifically targets the upstream conserved region 2 of PDE4, instead of its catalytic domain, and Eggmanone displays good pharmacokinetic properties [43, 44]. Future study of Eggmanone in treatment of AD dogs is warranted. Moreover, the increase of miR-203 in plasma of dogs is consistent with the previous study in serum of children with AD, highlighting similarities of AD in both dogs and humans [28]. MiR-203 is present in keratinocytes and has been shown to be increased in human psoriasis lesions [45], and its expression leads to inhibition of proliferation of epidermis stem cells and is involved in terminal differentiation of cells in the epidermis [46]. The increased expression of miR-203 in the plasma of AD dogs may be associated with down-regulation of the miR-203 target SOCS-3 [45]. It will be also of further interest to analyze miR-203 expression in the skin lesions of dogs with AD to determine if increased expression in the plasma correlates with a change of expression in serum. In addition, both PDE4A and miR-483 did not reach statistical significance which could be due to the limited sample size and further studies in a larger sample size may be needed in future.

In addition, controversial results of the CD4^+^/CD8^+^ T cell ratio were reported in association with AD dogs before [11–13, 15], and our result suggests a slight but not statistically significant increase of the CD4^+^/CD8^+^ ratio in AD dogs, which is in agreement with Beccati et al who showed no significant differences in the ratio of healthy dogs, atopic dogs and atopic dogs treated with cyclosporin. It is not surprising that the CD4^+^/CD8^+^ T cell ratio is not significantly changed in the blood of AD dogs as this ratio is usually associated with severe autoimmune disease (increase) or immune suppressive viral infections such as HIV or FIV (decrease), and the skin condition would have to be quite severe to affect the systemic T cell population [47, 48].

Furthermore, our study validates that the downregulation of three genes (PIAS1, RORA, SH2B1) are previously associated with canine AD in comparison to the healthy dogs. Interestingly, RORA was recently reported to involve in transactivation of IL-10 promoter [49], and downregulation of RORA may contribute to the decrease of serum IL-10 in our study. Finally, our cytokine profiling showed significantly elevated expression levels of Th2 inflammatory cytokines IL-13 and IL-31 as well as pro-inflammatory cytokine TNF-α, and dramatically decreased expression levels of TH1 cytokine IFN-γ and anti-inflammatory cytokine IL-10 in AD dogs. These results suggest that the Th2 response is increased and the Th1 response is decreased in the isolated PBMCs from AD dogs. Particularly, IL-31 (a TH2 cytokine) has attracted a lot of attention in recent years for its role in pruritus and atopic inflammation, and recent studies demonstrated serum IL-31 levels positively correlate with disease severity in both children and dogs with AD [50, 51]. Although total serum IgE levels are elevated in the humans with AD [52, 53], the values are of no clinical relevance in the AD dogs in this study, which is in agreement with previous reports [34, 35]. In addition, controversial results regarding immunosuppressive cytokine TGF-β1 expression have been shown in canine AD [5, 11, 33, 54]. Here, our study indicates that TGF-β1 expression was dramatically elevated in AD dog sera in comparison with healthy controls, and the reported variations of TGF-β1 expression could be attributed to the varying degree of severity of inflammation and pruritus in AD patients. Moreover, TGF-β1 is usually immunosuppressive and can block inflammatory reactions, and elevated levels might indicate a feedback loop in reaction to the inflammatory response.

Our study is the first to demonstrate association of expression of PDE4D and MiR-203 with canine AD. Due to the lack of an additional control group for non-atopic inflammatory skin diseases in this study, further investigations are warranted on whether these findings are unique for AD disease.

## Statistical analysis

All values are expressed as means + SE (standard error). Comparison of means was conducted using Prism non-parametric tests, and results were considered statistically significant if the p-value was <0.05.

## Supporting information

S1 Table Healthy age, sex, breed, spay/neuter. (DOCX)

S2 Table Atopic age, sex, breed, spay/neuter. (DOCX)
