# Supplementary material for: Phosphodiesterase 4D, miR-203 and selected cytokines in the peripheral blood are associated with canine atopic dermatitis
Source: PLoS One. 2019 Jun 21;14(6):e0218670. doi: 10.1371/journal.pone.0218670 (PMC6588236; doi:10.1371/journal.pone.0218670)
Supplement: S1 Table — (DOCX) [file pone.0218670.s001.docx]

**S1 Table: Healthy Age, Sex, Breed, Spay/Neuter**

| **Patient** | **Age (Year and Month)** | **Sex** | **Breed** | **Spay/Neuter** |
| --- | --- | --- | --- | --- |
| H1 | 9 Years | Female | Rat Terrier | Spay |
| H2 | 9 Years | Male | Chihuahua | Neuter |
| H3 | 2 Years and 5 Months | Female | Mixed | Spay |
| H4 | 1 Year and 2 Months | Male | Pitbull Mix | Neuter |
| H5 | 1 Year and 8 Months | Female | Plott Hound | Intact |
| H6 | 1 Year and 8 Months | Male | Plott Hound | Neuter |
| H7 | 2 Years | Female | Cattle Dog Cross | Spay |
| H8 | 2 Years | Male | Cattle Dog Cross | Neuter |
