# Supplementary material for: Phosphodiesterase 4D, miR-203 and selected cytokines in the peripheral blood are associated with canine atopic dermatitis
Source: PLoS One. 2019 Jun 21;14(6):e0218670. doi: 10.1371/journal.pone.0218670 (PMC6588236; doi:10.1371/journal.pone.0218670)
Supplement: S2 Table — (DOCX) [file pone.0218670.s002.docx]

**S2 Table: Atopic Age, Sex, Breed, Spay/Neuter**

| **Patient** | **Age (Year and Month)** | **Sex** | **Breed** | **Spay/Neuter** |
| --- | --- | --- | --- | --- |
| AD400 | 10 Years and 7 months | Female | Golden Retriever | Spay |
| AD500 | 5 Years and & 2 Months | Male | Miniature Pinscher Mix | Neuter |
| AD900 | 3 Years and 7 Months | Female | German Shepard | Spay |
| AD1000 | 6 Years and 8 Months | Male | Shih Tzu | Neuter |
| AD1200 | 5 Years and 10 Months | Female | Great Dane | Spay |
| AD1300 | 10 Years and 1 Month | Male | Cocker Spaniel | Neuter |
| AD1400 | 7 Years and 1 Month | Male | Boxer | Neuter |
| AD1500 | 8 Years and 1 Month | Male | Poodle | Neuter |
| AD1600 | 5 Years and 7 Months | Male | Terrier Mix | Neuter |
